# A participatory epidemiological and One Health approach to explore the community’s capacity to detect emerging zoonoses and surveillance network opportunities in the forest region of Guinea

**DOI:** 10.1371/journal.pntd.0010462

**Published:** 2022-07-11

**Authors:** Marie-Jeanne Guenin, Hélène Marie De Nys, Marisa Peyre, Etienne Loire, Suporn Thongyuan, Abdoulaye Diallo, Léonce Zogbelemou, Flavie Luce Goutard

**Affiliations:** 1 UMR ASTRE, Université de Montpellier, CIRAD, INRAE, Montpellier, France; 2 Faculty of Veterinary Medicine, Kasetsart University, Bangkok, Thailand; 3 UMR ASTRE, CIRAD, RP-PCP, Harare, Zimbabwe; 4 Direction Nationale des Services Vétérinaires, Conakry, République de Guinée; Swiss Tropical and Public Health Institute: Schweizerisches Tropen- und Public Health-Institut, SWITZERLAND

## Abstract

The Ebola virus disease epidemic that threatened West Africa between 2013 and 2016 was of unprecedented health magnitude. After this health crisis, studies highlighted the need to introduce community-based surveillance systems and to adopt a One Health approach. This study aimed to provide preparatory insights for the definition of a community-based surveillance system for emerging zoonoses such as viral hemorrhagic fevers in Guinea. The objective was to explore the disease detection capacity and the surveillance network opportunities at the community level in two pilot areas in the forest region of Guinea, where the epidemic emerged. Based on a participatory epidemiological and One Health approach, we conducted Focus Group Discussions with human, animal and ecosystem health actors. We used a range of participatory tools, included semi-structured interviews, ranking, scoring and flow diagram, to estimate the local knowledge and perception of diseases and clinical signs and to investigate the existing health information exchange network and its related strengths and weaknesses. The results showed that there is heterogeneity in knowledge of diseases and perception of the clinical signs among actors and that there are preferred and more effective health communication channels opportunities. This preparatory study suggests that it is necessary to adapt the case definitions and the health communication channels to the different actors who can play a role in a future community-based surveillance system and provides recommendations for future surveillance activities to be carried out in West Africa.

## Author summary

Viral hemorrhagic diseases have a high risk of emergence in tropical regions. The consequences on public health are often disastrous in the low- and middle-income countries that face difficulties to control epidemics. This scenario has unfortunately occurred in West Africa between 2013 and 2016 with the Ebola virus disease epidemic. There is an urgent need to increase the capacity for rapid detection of the emergence of such diseases and adopting a One Health approach since most of them are zoonotic. Community-based surveillance seems appropriate to address this issue in the context of these countries. We collected preparatory data through semi-structured interviews of community actors in human health, animal health and ecosystem health in Guinea. We used participatory epidemiology that appears to be a consistent method to assess local knowledge and perceptions of diseases and clinical signs and to identify health information exchange network opportunities. We aimed to provide relevant recommendations for the design of adapted case definitions and surveillance network in the prospect of implementing a community-based surveillance system of viral hemorrhagic fevers in Guinea and future surveillance activities to be carried out in West Africa.

## 1. Introduction

The emerging zoonotic diseases constitute threats to our modern world. Their rate of incidence is increasing, driven by anthropogenic factors such as international trade, human and animal populations movements and the disruption of ecosystems due to human activities; which are no more and no less than consequences of world population growth and globalization [1]. Nowadays, at least 75% of emerging diseases affecting humans are of animal origin and most of them originate in wildlife [2,3]. Several zoonotic diseases emerged from wildlife over these last decades such as the Nipah epidemic in 1999 in Malaysia, the Ebola virus disease (EVD) outbreak in West Africa in 2013 and more recently the COVID-19 pandemic in 2019 [4–8].

The EVD outbreak that occurred in West Africa between 2013 and 2016 was on a scale never seen before, with more than 20,000 reported cases and more than 11,000 deaths [9]. Governments have been overwhelmed by this health event. The surprising nature of this health disaster also lies in its geographical distribution. The EVD used to emerge in Central and East Africa. For the first time it appeared in West Africa with an index case retrospectively identified in Guinea and dated December 2013 [10]. Then, the EVD epidemic rapidly spread to other regions of Guinea and neighboring countries. Several factors led to a late response and a difficulty in containing the epidemic in Guinea: the presence of the ecological niche of the disease, susceptible populations, an insufficient response capacity, risky behaviors conducive to human-wildlife contacts and the community mistrust [11].

Poor regions of the world face many challenges such as a lack of health infrastructures, an insufficient access to health, communication problems and a lack of resources. This situation creates difficulties for the coordination of surveillance systems and the effective use of data [12]. Nowadays, participatory epidemiology is increasingly used for active surveillance of endemic, epidemic and emerging diseases. It consists of interactive participation to collect data, analyze them and plan action [13]. Community-based surveillance “is the systematic detection and reporting of events of public health significance within community by community members” [14]. It explores the social context in which a disease occurs and the host-agent-environment interactions. The participatory surveillance system, with the help of community workers, can therefore be a solution to make monitoring possible by reducing the burden on health infrastructures and supporting data collection [15].

To avoid a future pandemic, a One Health approach is a key strategy for global health security [16]. The tripartite collaboration, included the World Health Organization (WHO), World Organization for Animal Health (OIE) and Food and Agricultural Organization (FAO), called for a One Health surveillance of diseases and recommend to coordinate and address health risk at the human-animal-ecosystems interfaces [17]. In November 2020, the United Nations Environment Programme (UNEP) joint this tripartite collaboration in a new international expert panel to address the emergence and spread of zoonotic diseases [18]. This panel defined the One Health as “an integrated, unifying approach that aims to sustainably balance and optimize the health of people, animals and ecosystems” and recognized these three sectors of health as “closely linked and inter-dependent” [19]. Several studies support the hypothesis that a One Health approach provides a positive effect for emerging infectious diseases prevention [20–22]. By combining knowledge on animals, humans and ecosystems, surveillance and detection strategies are strengthened [23].

The purpose of our study was to provide preparatory insights for the definition of a community-based surveillance system in Guinea for the early detection of emerging zoonoses and viral hemorrhagic fevers (VHFs) in particular; as they have a high pandemic and outbreak receptivity in this area [24]. We used a participatory epidemiological and One Health approach and conducted Focus Group Discussions (FGDs) with human, animal and ecosystem health actors to estimate local knowledge and perception of diseases and clinical signs. We also investigated the existing health information exchange network and its related strengths and weaknesses.

## 2. Material and methods

### 2.1 Ethics statement

Official authorization from the National Department of Veterinary Services and at the prefectural level allowed us to work on these areas. The study was validated by the National Health Research Ethics Committee of Guinea (046/CNERS/18, 023/CNERS/19). Respondents participated freely and anonymously to the research study. The project was presented and translated before each interview so that participants could knowingly sign a consent form.

### 2.2. Study area

**Fig 1:**
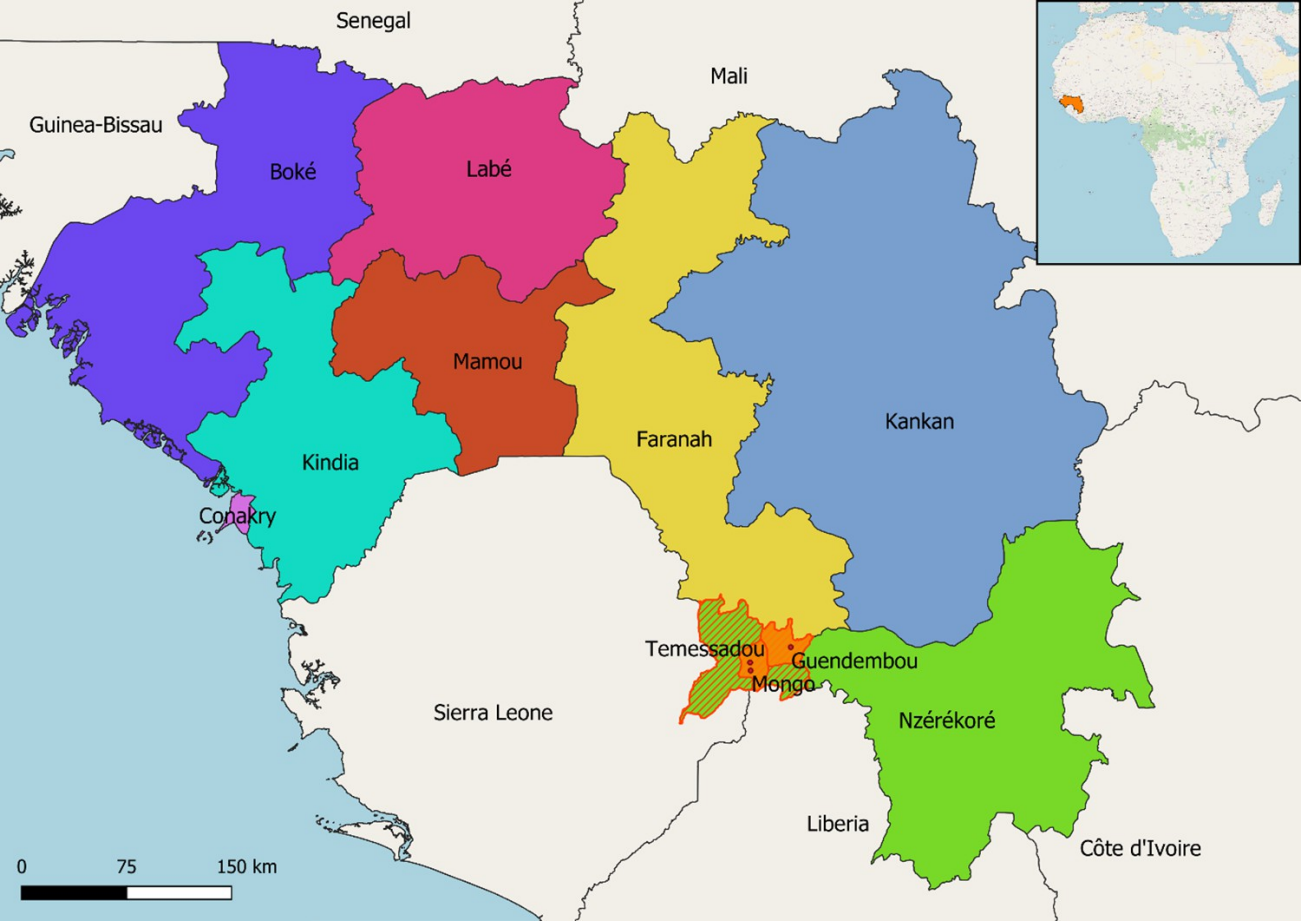
Map of the Republic of Guinea and location of the study areas. The study areas were located in the sub-prefectures of Guendembou and Temessadou (orange area) in the prefecture of Guéckédou (orange hatched area) in the N’Zérékoré region (green area). Focus Group Discussions were conducted in the villages of Guendembou, Temessadou and Mongo (red dots). Map created using the Free and Open Source QGIS software (https://www.qgis.org) and OpenStreetMap (https://www.openstreetmap.org) and GADM (https://www.gadm.org) geographic databases.

The study was conducted in the prefecture of Guéckédou in the forest region of N’Zérékoré in Guinea. Based on a previous study on the socio-cultural and economic practices increasing the risk of zoonotic transmission from wildlife and the community’s perception of One health surveillance and in consultation with the National Direction of Veterinary Services (Direction Nationale des Services Vétérinaires) and key resource persons, we selected the two sub-prefectures of Guendembou and Temessadou, for their close human-domestic animal-wildlife interface and for the presence of human, animal and ecosystem health actors [25]. We conducted FGDs in their respective chief towns and in the village of Mongo (Fig 1).

### 2.3 Participants

We conducted FGDs with human, animal and ecosystem health actors to estimate local knowledge and perception of diseases and clinical signs and to investigate the existing health information exchange network and the potential collaborations for community-based surveillance.

From the human health sector, we identified women and Community Human Health Workers (CHHWs). We decided to have non-mixed groups of women to ensure their representativeness because they are under-represented among other categories of actors. Women are involved in the care of family members and, as such, can play a role in disease detection. CHHWs are volunteers located in village and elected by the community. They are literate and specially trained in birth control, malaria treatment, awareness of diseases and surveillance. They routinely report human health information to the health center.

From the animal health sector, we identified breeders and Community Animal Health Workers (CAHWs). CAHWs are volunteers located at the district level and elected by the community. They are literate and specially trained in conflict management between plant growers and animal breeders, veterinary treatment, awareness of animal diseases and surveillance.

From the ecosystem health sector, we identified hunters, Community Informants (CIs) and rangers. In this study area, traditional hunting is practiced by hunters from the Kissi ethnolinguistic group. CIs are volunteers from the community located at the district level. They are literate and elected by the sub-prefectural Water and Forest Department to report on bushfires and non-compliance with hunting regulation and conservation activities. The rangers are located at the sub-prefectural level and work for the Water and Forest Department which is the most decentralized service of the Ministry of Environment. They control and manage all activities related to the environment and conservation such as fishing, hunting, deforestation, wildlife monitoring.

### 2.4 Data collection

#### 2.4.1 Participatory epidemiological approach

The participatory epidemiology is a bottom-up approach based on active participation of individuals in defining their own solutions tailored to their development stakes. This method is used to collect and analyze qualitative epidemiological information from local knowledge and expertise and is consistent to increase involvement of communities in the design and implementation of animal health program such as surveillance systems [13,26]. In this present study we investigated the perception of the disease and clinical signs to assess the case definition adaptability [27]. Participatory epidemiology is also interesting to explore the health information exchange network opportunities as it promotes local initiative and communication between the various actors [28]. Data were collected by an investigation team including a veterinarian from the National Direction of Veterinary Services, the Livestock Prefectural Director of Macenta in N’Zérékoré forest region and two French veterinary students. The investigation team was trained to conduct semi-structured interviews (SSIs), to use participatory tools and to record data in an appropriate format for analysis. For each FGD, we distributed roles among the investigation team to have one facilitator, one analyst and two note-takers. We conducted the FGDs in French or in the local language and literate persons from the sub-prefectures translated from Kissi into French.

#### 2.4.2 Data triangulation and sampling method

We applied data triangulation as main sampling criteria to ensure data reliability and saturation [29–31]. We expected between eight and ten participants per FGD to ensure data quality without compromising participation and facilitation. In each sub-prefecture, the head of the health center, the official veterinarian and the head of the Water and Forest Department selected the participants from the human, animal and ecosystem health sectors respectively. Participation to the FGDs was without gender restriction, except for the non-mixed groups of women, and we voluntarily included heads of breeders and heads of hunters to investigate their role in the existing animal and ecosystem health information exchange network. We conducted two sessions of FGDs in each sub-prefecture and expected as many of the participants as possible to be the same from one session to the other to foster their understanding of the research activities.

#### 2.4.3 Data on local knowledge and perception of diseases and clinical signs

Between February and March 2019, we conducted a first session of FGDs with each type of participants in both sub-prefectures to estimate local knowledge and perception of disease and clinical signs in the human, domestic animal and wildlife populations. We used several participatory tools as described by Catley et al. [32] such as SSIs, ranking and scoring. First, we asked participants to list the clinical signs, and their related diseases, they observe in the human, domestic animal or wildlife populations, and we noted them on post-it notes. Secondly, to reduce the large number of clinical signs and ensure the feasibility of the following steps, we asked them to select those they perceive as life-threatening or as a zoonotic risk. Then, we asked them to rank these selected clinical signs from the most to the least frequently observed. Finally, we asked participants to score these same and previously selected clinical signs by distributing one hundred beans proportionally to the associated threat regarding their own health; even for the signs they observe in the animal populations.

#### 2.4.4 Data on the health information exchange network

The first session of FGDs allowed us to collect qualitative data on community’s response to health threats and human, animal and ecosystem health actors’ roles. In April 2019, we conducted a second session of FGDs in each sub-prefecture to gather women and CHHWs, breeders and CAHWs and hunters and CIs. We used flow diagrams to explore the existing health information exchange network, by drawing the interactions between actors on a poster, and to discuss its related strengths and weaknesses and better alternatives for an efficient community-based surveillance of emerging zoonotic diseases [33].

### 2.5 Data analysis

All the FGDs were recorded and transcribed on Microsoft Word. We performed a thematic content analysis to extract the qualitative data from the transcripts and sort them in different broad themes on Microsoft Excel. Using a deductive approach, we identified the pre-conceived themes: stakeholders’ roles, local knowledge on diseases and detection capacity, communication of health information, and One Health collaboration [34]. Data were separately analyzed for each One Health sector.

#### 2.5.1 Qualitative analysis of data on local knowledge of diseases

Data on diseases and clinical signs were reported into a Microsoft Excel table to generate diseases-clinical signs matrix. We extracted from the matrix the commonly listed diseases, the diseases under surveillance, the VHFs and the zoonotic diseases. To estimate participants’ knowledge, we compared the clinical picture they used to describe the diseases under surveillance with the clinical pictures cited in the OIE and WHO descriptions and with the case definitions provided to the CAHWs and CHHWs [35–46].

#### 2.5.2 Analysis of rankings and scorings

Semi-quantitative data obtained from ranking and scoring were reported into a database and processed for analysis using graphical representations on Microsoft Excel. For each group of participants, we generated scatter plots of the signs that were ranked and scored to position them according to participants’ perception in terms of frequency of observation and concern regarding their own health. Then we plotted axes through the medians of the ranks and scores to generate four-way matrix: rare/frequent and health-threatening/not threatening. This method allowed to identify the clinical signs perceived as the rarest and the most health-threatening and to estimate participants’ perception of the clinical signs included in the human case definitions recommended by the WHO for Ebola or Marburg virus disease surveillance and in the animal case definition recommended by the FAO for the syndromic and participatory surveillance of the Rift Valley fever (RVF) which is one of the VHFs causing disease in animals [35,47].

#### 2.5.3 Analysis of data on the health information exchange network

From the flow diagrams we identified the actors who collect health information and the communication channels they use. We compiled all the communication channels drawn by the different groups of participants in a generic flow diagram. We performed a qualitative analysis of the FGDs to identify the actor’s constraints, reluctance and preferences to use these communication channels. The FGDs were also an opportunity for the participants to explore better alternatives for a more efficient communication of health information.

## 3. Results

### 3.1 Participation in the FGDs

For the first session of FGDs, we conducted twelve collective SSIs, six in the sub-prefecture of Guendembou and six others in the sub-prefecture of Temessadou. We recorded one-hundred-twelve participations. We identified the CIs after a first fieldwork in Guendembou and we included them in subsequent FGDs. For the second session, we conducted six FGDs, three in Guendembou and three in Temessadou. We recorded a fifty-two participation rate. The rangers were not included in this second session of FGDs because we preferred to have actors as close to the community as possible to complete information (Table 1). The average number of participants per FGDs was of 9.3 for the first session and of 8.7 for the second one.

**Table 1:** Number and type of participants per Focus Group Discussions.

Focus Group Discussions		First session			Second session		
Participants		Area 1	Area 2	Total	Area 1	Area 2	Total
Human health	Women	14	9	23	6	5	11
	CHHWs	9	11	20	5	5	10
Animal health	Breeders	10	7	17	3	6	9
	CAHWs	8	12	20	1	5	6
Ecosystem health	Hunters	11	12	23	8	5	13
	CIs	Unidentified	2	2	0	3	3
	Rangers	4	3	7	Not included		-
Total		56	56	112	23	29	52

Area 1, sub-prefecture of Guendembou; Area 2, sub-prefecture of Temessadou; CHHWs, Community Human Health Workers; CAHWs, Community Animal Health Workers; CIs, Community Informants.

### 3.2 Capacity of the communities to detect human diseases

#### 3.2.1 Local knowledge about human diseases

The disease-clinical signs matrix showed knowledge heterogeneity among groups of participants on diseases and their associated clinical pictures (Matrix A in S1 Matrix). Both groups of CHHWs cited seven of the eight diseases under surveillance for which they received a technical sheet with case definitions. Among these diseases under surveillance, they commonly mentioned the VHFs such EVD and yellow fever and CHHWs from Temessadou mentioned the Lassa fever as well. Women from Guendembou mentioned four of the eight diseases under surveillance for which they were sensitized through awareness campaign. Among these four diseases they mentioned two VHFs such as EVD and another one they named “nasal hemorrhagic disease”. In comparison, women from Temessadou did not mentioned any disease under surveillance and VHFs. None of the groups mentioned the five other diseases under surveillance for which CHHWs did not receive technical sheet with case definitions (Table 2).

**Table 2:** Clinical pictures associated with human diseases known by women and Community Human Health Workers.

Group of participants			CHHWs				Women		
			Area 1		Area 2		Area 1		Area 2
Diseases under surveillance	**VHFs**	**EVD**	0		Hematemesis	✓	0		-
		**Lassa fever**	-		Hematemesis	✓✓	-		-
		**NHF**	-		-		Bleeding	✓	-
							Headache	✓	
		**Yellow fever**	Red eyes	✓	Yellow eyes	✓✓	-		-
			Yellow urine	✓	Yellow urine	✓			
					Anorexia	✓			
					Bloating				
					Breathing difficulties				
					Weight loss				
	**Not VHFs**	**Measles**	Pimples	✓✓	Pimples	✓✓	Pimples	✓✓	-
							Fever	✓✓	
							Yellow eyes		
		**Meningitis**	Neck stiffness	✓✓	Neck stiffness	✓✓	-		-
		**Cholera**	Diarrhea	✓✓	Diarrhea	✓✓	Diarrhea	✓✓	-
			Deep-set eyes		Vomiting		Vomiting		
		**Poliomyelitis**	Paralysis	✓✓	Paralysis	✓✓	-		-
		**Neonatal tetanus**	Convulsions	✓	-		-		-
Diseases not under surveillance	**Common**	**Malaria**	Coma		Coma				
			Convulsions		Convulsions				Convulsions
			Fever		Fever		Fever		Fever
			Vomiting		Vomiting				Vomiting
					Headache		Headache		Headache
					Vertigo				Vertigo
			Loss of consciousness		Nausea				Diarrhea
					Pallor				
		**Tuberculosis**	Cough		Hematemesis		Cough		-
					Weight loss		Breathing difficulties Fatigue		
		**Anemia**	Palmar pallor		Pallor		Pallor		-
			White eyes				Dehydration		
		**Child malnutrition**	Bloating		Growth delay		-		-
			Edema						
			Weight loss						
		**Tension**	-		-		0		Paralysis
		**Asthma**	-		-		Breathing difficulties		Breathing difficulties
							Cough		
							Fatigue		
		**Opimo**	-		-		Headache		Headache

Area 1, sub-prefecture of Guendembou; Area 2, sub-prefecture of Temessadou; CHHWs, Community Human Health Workers; VHFs, viral hemorrhagic fevers; EVD, Ebola virus disease; NHF, nasal hemorrhagic fever; Opimo, intracranial vascular hypertension; green tick symbol, clinical signs in case definitions provided to the Community Human Health Workers; orange tick symbol, clinical signs in WHO descriptions of diseases; yellow underlined text is the clinical signs commonly cited by at least two groups of participants.

The VHFs are poorly described. CHHWs from Temessadou associated hematemesis with Lassa fever and EVD but did not associate fever, other signs of hemorrhage or mortality which are including in the VHFs case definition. CHHWs and women from Guendembou did not associate any signs with EVD. Women from Guendembou associated bleeding and headache to the “nasal hemorrhagic disease”. Among the signs included in the specific yellow fever case definition, only CHHWs from Temessadou associated the yellow eye sign. None of CHHWs groups associated fever and yellow skin but they associated other signs included in the WHO definition such yellow urine. The other diseases under surveillance are better and more homogeneously described, except by women from Temessadou. The groups that mentioned these diseases associated at least one of the signs including in case definition, except for neonatal tetanus. CHHWs from both sub-prefectures did not associate fever to measles and bulging fontanelle in children and fever to meningitis. Seven diseases which are not under surveillance were listed by at least two groups of participants. The groups associated at least one common clinical sign. Malaria is the only disease mentioned and similarly described by all groups of participants. Except the group of women from Guendembou, all the groups described the disease with five to seven signs. This disease is subject to a specific health program and is frequently observed in the community, as mentioned by a CHHW from Temessadou: “Malaria is very common: vomiting, fever, headache”. Women from Guendembou also identified diseases they suffer from or they observe in the community: “All of those [women] sitting here are all sick. Tension, filariasis, malaria are spread in this area.”.

#### 3.2.2 Ranking and scoring of clinical signs in human health

CHHWs from Guendembou listed twenty clinical signs or symptoms, selected seventeen of them as health-threatening, and ranked and scored these seventeen signs. CHHWs from Temessadou listed thirty-three signs, selected twenty-seven of them as health-threatening, they only ranked the eighteen most frequently observed signs but did not rank the nine signs identified as the rarest. They added four signs during the scoring (appendicitis, epilepsy, hernia, hydrocele) and scored thirty-one signs in total. Women from Guendembou listed twenty-two signs, selected eleven of them as health-threatening and ranked these eleven signs. They added one sign (joint pain) during the scoring and scored twelve signs in total. Women from Mongo, sub-prefecture of Temessadou, listed thirty-six signs and selected twenty-five of them as health-threatening. They did not rank these clinical signs but divided them into two categories: nineteen frequently observed signs and six rarely observed signs. They scored these twenty-five signs.

**Fig 2:**
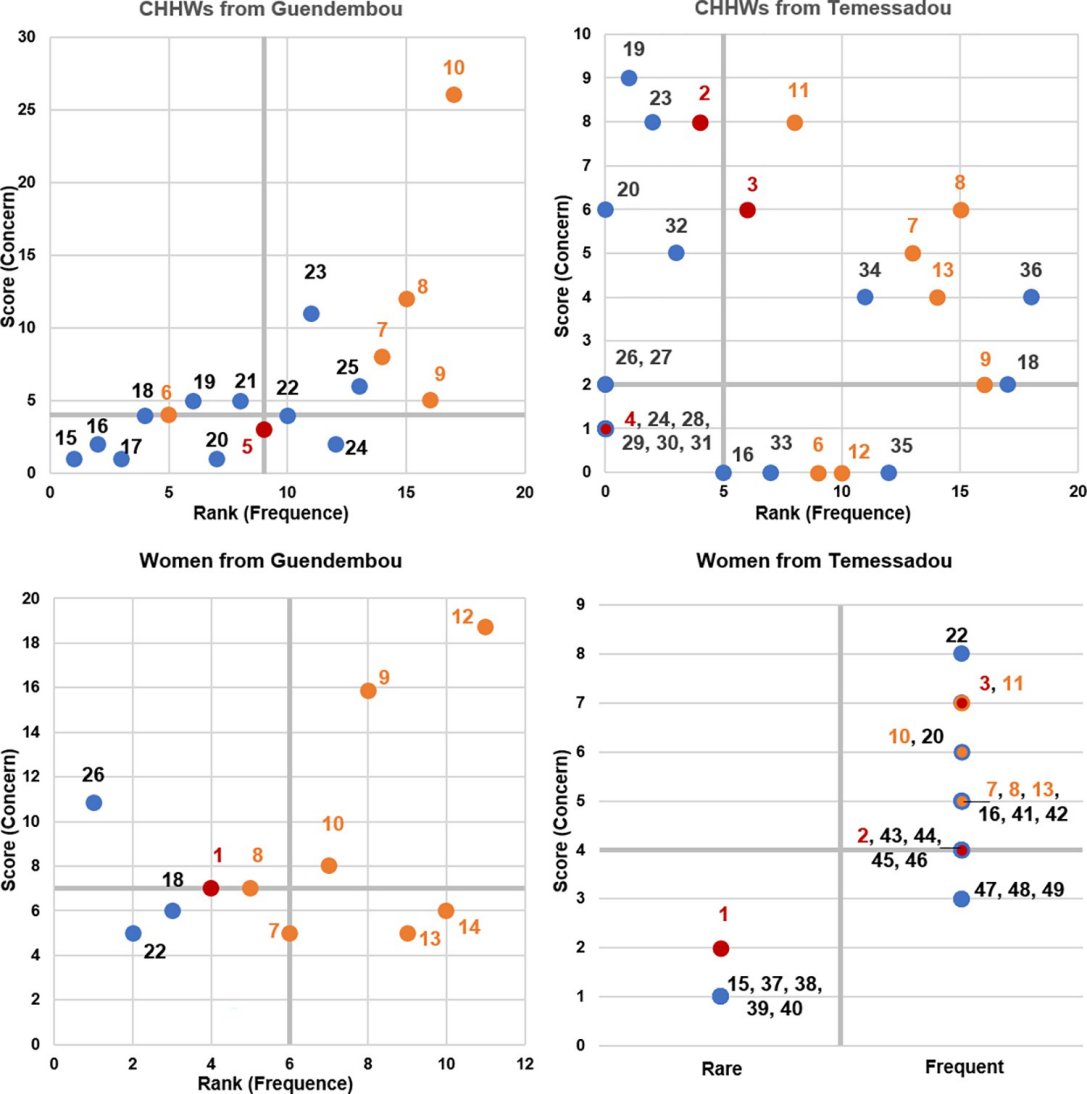
Perception of the signs ranked and scored according to rarity and concern by Community Human Health Workers and women from the community. On the x-axis: rank associated with the frequency of observation of the sign, from the least to the most frequent. On the y-axis: score associated with the threat for human health, from the least to the most health threatening. Axes are cut at their median. Red dots represent hemorrhagic signs: 1. Bleeding 2. Hematemesis 3. Bloody stools 4. Hematuria 5. Red eyes and dark yellow urine. Orange dots represent signs included in the WHO case definitions for viral hemorrhagic fever surveillance: 6. Anorexia 7. Diarrhea 8. Vomiting 9. Headache 10. Fever 11. Breathing difficulties 12. Fatigue 13. Abdominal pain 14. Muscle pain. The blue dots represent all other signs that are ranked and scored as not fitting these categories: 15. Paralysis 16. Bloating 17. Edema 18. Pimples 19. Loss of consciousness / coma 20. Neck stiffness 21. Palmar pallor 22. Cough 23. Convulsions 24. Weight loss 25. Deep-set eyes 26. Yellow eyes 27. Yellow urine 28. Pimples located on one side of the body 29. Mutilation 30. Weight gain 31. Polyuria 32. Malnutrition 33. Pyuria 34. Pallor 35. Rectal prolapse 36. Wound 37. Epilepsy 38. Loss of speech 39. Vertigo 40. Gbassama 41. Taeniasis 42. Abortion 43. Constipation 44. Opimo 45. Breast pain 46. Swelling 47. Chest pain 48. Toothache 49. Absence of menstrual period. Women from Temessadou did not assign a rank to the signs mentioned but classified them into two categories: rare and frequent.

Most of the VHFs clinical signs are perceived as frequent and health-threatening but the specific hemorrhagic signs are heterogeneously ranked and scored by the groups of participants. Among all the hemorrhagic signs mentioned, and which are of interest for the detection of VHFs, the ones which are perceived as rare are: bleeding by women from the two areas, red eyes by CHHWs from Guendembou, hematemesis and hematuria by CHHWs from Temessadou; and the ones which are perceived as threatening are: bleeding by women from Guendembou, bloody stools and hematemesis by women and CHHWs from Temessadou. A CHHW and a woman from Temessadou particularly said: "If you vomit blood, you will die.". Bleeding, hematemesis and hematuria are the only hemorrhagic signs perceived as both rare and threatening. Other non-pathognomonic signs which are of interest for the detection of VHFs, particularly during outbreak, were ranked and scored. Most of them are perceived as frequent, except, anorexia by CHHWs from Guendembou and vomiting and diarrhea by women from Guendembou. The ones which are perceived as health-threatening are: headache, vomiting, fever, breathing difficulties by the groups that scored them. Diarrhea is perceived as health-threatening by all the groups, except women from Guendembou. Abdominal pain is perceived as health-threatening by CHHWs and women from Temessadou and as not threatening by women from Guendembou. Muscle pain is not perceived as threatening. Anorexia is perceived as threatening by CHHWs from Guendembou and as not threatening by CHHWs from Temessadou. Fatigue is perceived as threatening for women from Guendembou and not for the CHHWs from Temessadou (Fig 2).

### 3.3 Capacity of the communities to detect animal diseases

#### 3.3.1 Local knowledge about animal diseases

The disease-clinical signs matrix showed knowledge heterogeneity among groups of participants on diseases and their associated clinical pictures (Matrix B in S1 Matrix). Most of the diseases mentioned by CAHWs and breeders are observed in livestock. Some of them worry them because of the zoonotic risk, as mentioned by a breeder from Guendembou: “Scabies is also a worrying disease. The consumption of contaminated meat is a serious risk to human health.”. Other diseases worry them because of the economic loss they generate, as a breeder from Temessadou mentioned: “Animal diseases affect our moral because of their negative economic impact.”. CAHWs from Guendembou mentioned four of the five diseases under surveillance for which they received technical sheets with case definitions. CAHWs from Temessadou mentioned three of them. Breeders from Guendembou and from Temessadou mentioned two of the diseases under surveillance for which they were sensitized.

Among the zoonotic diseases under surveillance, all the groups mentioned anthrax whereas this disease is not prevalent in the study area as mentioned by a breeder from Guendembou: “We have never observed case of anthrax but we know this disease.”. They did not mention the hepatic lesion used in the case definition but they associated other signs provided in the OIE definition such as bleeding and mortality by the CAHWs and the breeders respectively. CAHWs from both sub-prefectures commonly mentioned rabies. They associated the clinical picture provided in the case definition but also associated other signs which are not described in the OIE technical disease card. Rabies is prevalent in our study area as mentioned by a CAHW from Guendembou: “Rabies affects dogs and is very common in our country.”. CAHWs from Temessadou and breeders from Guendembou mentioned avian influenza. They did not associate the nasal discharge sign cited in the case definition but breeders from Guendembou associated mortality. CAHWs from Guendembou and breeders from Temessadou mentioned other diseases under surveillance which are not zoonotic. They commonly mentioned the New Castle disease and CAHWs from Guendembou mentioned the PPR as well. They did not associate signs to the PPR. They described the New Castle disease with signs not included in case definition, except dirty hind legs. Some of these signs are part of the OIE clinical picture such falling wings, fatigue and mortality. CAHWs from Guendembou mentioned two additional zoonotic diseases which are not under surveillance such scabies and tuberculosis. The breeders from Guendembou also mentioned scabies but did not associate clinical signs. Three other diseases which are not under surveillance and not zoonotic were listed by at least two groups of participants. Hair loss and diarrhea were commonly used to define eczema and parasitosis respectively (Table 3).

**Table 3:** Clinical pictures associated with animal diseases known by Community Animal Health Workers and breeders.

Group of participants			CAHWs				Breeders			
			Area 1		Area 2		Area 1		Area 2	
**Diseases under surveillance**	**Zoonotic**	**Rabies**	Aggressiveness	✓✓	Aggressiveness	✓✓	-		-	
			Photophobia		Salivation	✓✓				
			Animal turning on itself		Animal turning on itself					
			Hydrophobia							
		**Anthrax**	Bleeding	✓	Bleeding	✓	Mortality	✓	Mortality	✓
			Bloating	✓	Bloody stools	✓			Black meat	
			Black blood		Fall	✓				
					Tremors	✓				
					Salivation					
					Black body part					
		**Avian influenza**	-		0		Mortality	✓	-	
	**Not zoonotic**	**PPR**	0		-		-		-	
		**New Castle disease**	Dirty hind legs	✓	-		-			
			Falling wings	✓					Fatigue	✓
			Nasal discharge						Mortality	✓
			Salivation						Salivation	
			Tearing							
**Diseases not under surveillance**	**Zoonotic**	**Scabies**	Hair loss		-		0		-	
			Presence of lice or ticks							
			Wound							
		**Tuberculosis**	Cough		-		-		-	
	**Common**	**Foot rot**	Hoof wound		-		0		Excessive hoof growth	
			Lameness							
		**Eczema**	-		Hair loss		-		Hair loss	
									Crusts	
									Hard skin	
									Itching	
		**Parasitosis**	-		Diarrhea		Diarrhea		-	
							Bloating			

Area 1, sub-prefecture of Guendembou; Area 2, sub-prefecture of Temessadou; CAHWs, Community Animal Health Workers; green tick symbol, clinical signs in case definitions provided to the Community Animal Health Workers; orange tick symbol, clinical signs in OIE descriptions of diseases; yellow underlined text is the clinical signs commonly cited by at least two groups of participants.

#### 3.3.2 Ranking and scoring of clinical signs in animal health

CAHWs from Guendembou listed twenty-five signs, selected ten of them as threatening for their own lives, ranked and scored these ten signs. CAHWs from Temessadou listed twenty-nine signs, selected sixteen of them as threatening for their own lives, ranked and scored these sixteen signs. Breeders from Guendembou listed seventeen signs, selected fifteen of them as threatening for their own lives, ranked and scored these fifteen signs. Breeders from Temessadou, listed twenty-eight signs and selected fourteen of them as threatening for their own lives, ranked and scored these fourteen signs.

**Fig 3:**
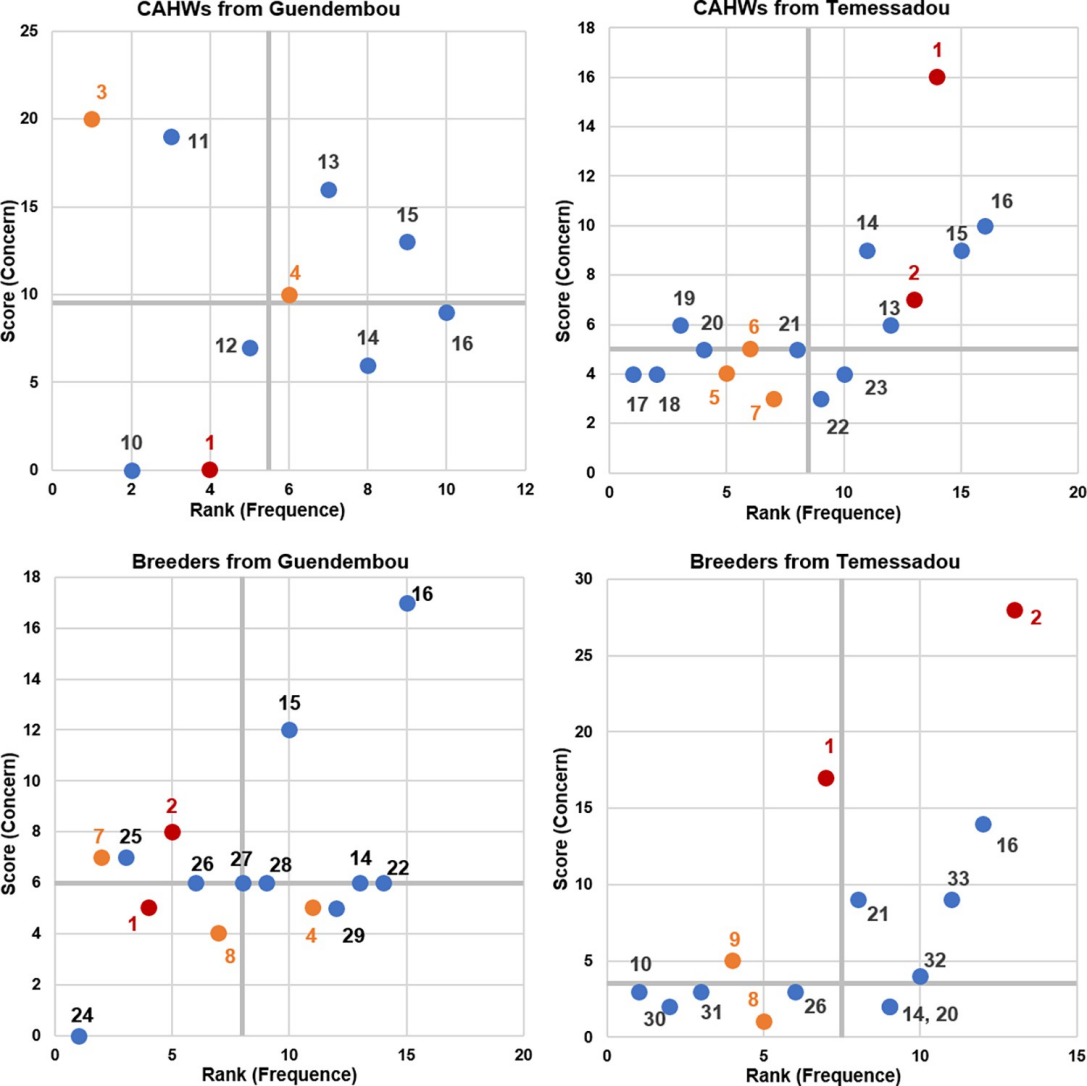
Perception of the signs ranked and scored according to rarity and concern by Community Animal Health Workers and breeders. On the x-axis: rank associated with the frequency of observation of the sign, from least to most frequent. On the y-axis: score associated with the concern for human health, from the least to the most threatening. Axes are cut at their median. Red dots represent signs included in case definitions for Rift Valley Fever surveillance: 1. Mortality 2. Abortion. Orange dots represent other non-pathognomonic signs of Rift Valley Fever: 3. Bleeding and black blood 4. Nasal discharge 5. Oral bleeding 6. Bloody stools 7. Fatigue 8. Anorexia 9. Bleeding. The blue dots represent all other signs that are ranked and scored as not fitting these categories: 10. Aggressiveness 11. Animal turning on itself, photophobia and hydrophobia 12. Falling wings and tearing in poultry 13. Hair loss 14. Salivation 15. Cough 16. Diarrhea 17. Black body part 18. Vulvar wound 19. Presence of worms in the flesh 20. Tremors and fall 21. Presence of flies 22. Bloating 23. Presence of ticks 24. Presence of venom in eyes 25. Malformation 26. Weight loss 27. Scabies 28. Cold 29. Fatigue in poultry populations 30. Presence of abdominal fat 31. Black meat 32. Hair loss, hard skin, itching and pimples 33. Cough and white lungs.

The Fig 3 showed a heterogeneous perception of the clinical signs among groups. Abortion and mortality, which are included in case definition for the surveillance of RVF are perceived differently among groups of participants. Abortion is perceived as frequent by CAHWs and breeders from Temessadou, as rare by breeders from Guendembou and as health-threatening by these three groups. We learned that brucellosis prevailed in this region and cause abortion in livestock. Mortality is perceived as rare by all groups except by CAHWs from Temessadou and as threatening for CAHWs and breeders from Temessadou and not threatening by CAHWs and breeders from Guendembou. Other non-pathognomonic signs of RVF were mentioned, ranked and scored. They are all perceived as rare except nasal discharge by CAHWs and breeders from Guendembou. Hemorrhagic signs of interest such as bleeding and bloody stools are perceived as threatening and oral bleeding as not threatening. Fatigue is perceived as threatening by breeders from Guendembou and as not threatening by CAHWs from Temessadou. Anorexia is perceived as not threatening by breeders from Guendembou and Temessadou.

### 3.4 Capacity of the communities to detect wildlife diseases

#### 3.4.1 Local knowledge about wildlife diseases

Forest rangers, hunters and CIs from both sub-prefectures did not mention any disease which affects wildlife populations but since the EVD outbreak they understood the zoonotic risk, as a ranger of Temessadou mentioned: “This is when Ebola emerged from the monkeys, from the bats, that we understood the zoonotic risk. Before we didn’t know. They constitute vital animal proteins but now we are very afraid to consume them.”. They defined a sick animal as a living animal that seems tired and presents an unusual behavior or a dead animal for no apparent reason. Hunters and CIs from Temessadou identified clinical signs or lesions on carcasses that they perceived as an indicator of disease occurrence but they did not associate these signs with a disease but rather with animal species (palm rat, mouse, bat, doe, agouti, etc.).

#### 3.4.2 Ranking and scoring of clinical signs in wildlife

**Fig 4:**
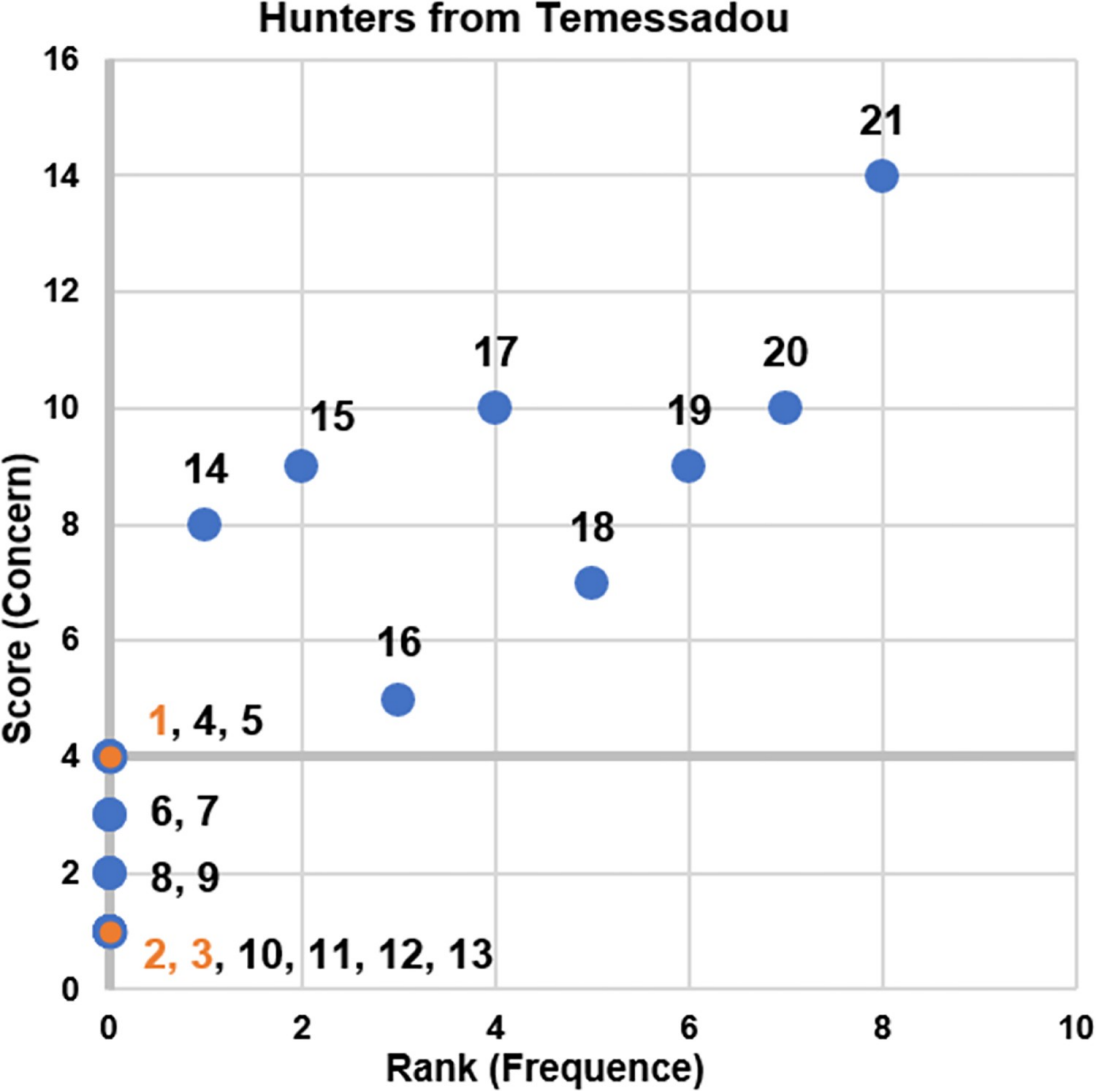
Perception of the signs ranked and scored according to rarity and concern by hunters from Temessadou. On the x-axis: rank associated with the frequency of observation of the sign, from least to most frequent. On the y-axis: score associated with the concern for human health conferred by these signs, from the least to the most threatening. Axes are cut at their median. Orange dots represent non-pathognomonic signs of Rift Valley Fever: 1. Nasal discharge 2. Fatigue 3. Yellow organs. Blue dots represent all signs that are ranked and scored: 4. Presence of water in the body 5. Black blood 6. Tearing 7. Immobility 8. Adhesion of organs 9. Absence of blood 10. Atrophied organs 11. Facial hypertrophy 12. Malformation 13. Hypotrophy of gall bladder 14. Presence of worms in liver 15. Presence of external maggots 16. Presence of red worms in stomach 17. Cough 18. Red wounds 19. Weight loss 20. Hair loss 21. Diarrhea.

Only hunters from Temessadou listed twenty-one signs and selected all of them as threatening for their own lives. They ranked the eight most frequently observed signs but did not rank the thirteen signs identified as the rarest. They scored these twenty-one signs. Among signs of RVF they mentioned nasal discharge, fatigue and yellow organs which are perceived as rare and only nasal discharge is perceived as threatening (Fig 4). They did not list mortality but they said that it is very rare to see several dead animals in the same area (“We have never seen an epidemic that kills many wild animals.”). The most threatening signs are the ones perceived as frequent. Some of these signs are visible at the autopsy such as presence of worms in liver, presence of water in the body, black blood, presence of red worms in stomach.

### 3.5 Health information exchange network

**Fig 5:**
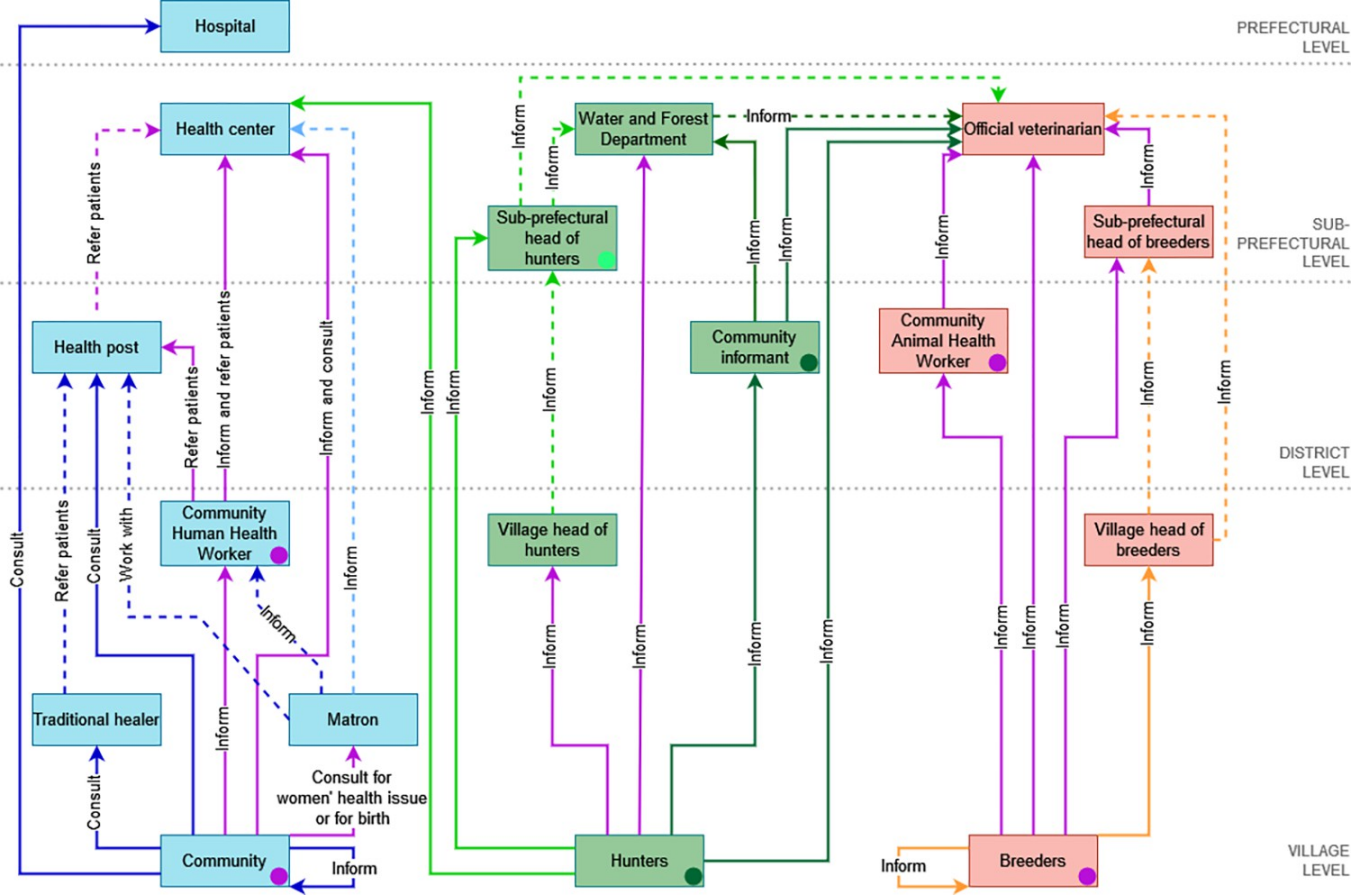
The community health information exchange network including human, animal and ecosystem health actors. Blue boxes: human health actors; green boxes: ecosystem health actors; red boxes: animal health actors; blue arrows: human health communication channels identified by actors from Guendembou (light blue) or Temessadou (dark blue); green arrows: ecosystem health communication channels identified by actors from Guendembou (light green) or Temessadou (dark green); orange arrows: animal health communication channels identified by actors from Guendembou; purple arrows: health communication channels commonly identified by actors from Guendembou and Temessadou; dashed arrows: health communication channels identified but not triangulated by actors; circle: actors from both sub-prefectures (purple) or ecosystem health actors from Guendembou (light green) or from Temessadou (dark green) who communicate health information to local authorities.

The Fig 5 shows that there are several opportunities to communicate health information from the village level to the sub-prefectural relevant actors in each health sector.

#### 3.5.1 Human health communication channels

In both sub-prefectures, when someone shows signs of disease, community members can inform the CHHW or directly inform or consult the health center. Women specifically consult matrons in case of women’s health issues or for birth. In Temessadou, community members also inform their community or consult the hospital, through ignorance of CHHW’s role, or traditional healers, mainly for financial reasons. According to CHHWs, the traditional healers and matrons may withhold information as mentioned by CHHWs: “The communication between matrons and CHHWs is only for birth issue. Otherwise, matrons generally do not transmit information to the CHHWs.”, “Healers only communicate with CHHWs when they do not manage to cure patients”. CHHWs from Guendembou and Temessadou refer patients to the health post or health center and inform the health center if there is a suspected case. In general, the community members trust the CHHW that they elected, but there is still some reluctance to inform him, as mentioned by a CHHW from Temessadou: “When a CHHW comes to provide free care to a person with malaria, people in village still mistrust the CHHW and say that CHHWs administer drugs that kills them.”.

#### 3.5.2 Animal health communication channels

In both sub-prefectures, breeders generally inform the official veterinarian or the head of breeders. Breeders can inform the CAHW but some of them are still reluctant towards CAHWs as mentioned: “There is reluctance. Even if a breeder sees his animal in a dilapidated state, he does not care. For him we ca not treat the animal.”. Some breeders do not want the veterinarian to be informed because of financial reasons as mentioned by a CAHW: “Sometimes the breeders do not have money and they are afraid to not be able to pay the veterinarian.”. In Temessadou breeders can also inform the animal’s owner or the head of breeders at the village level. CAHWs from Gendembou and Temessadou always inform the official veterinarian.

#### 3.5.3 Ecosystem health communication channels

At the community level, there is no surveillance system to report suspected case in the wildlife population. Nevertheless, the ecosystem health actors want to participate in the surveillance and there are existing communication channels to share information on species conservation, hunting management and forestry activities. In both sub-prefectures, hunters can directly inform the Water and Forest Department but they prefer communicate with their representative in the village because of the economic loss due to the carcass confiscation by rangers. For the same reason, hunters from Temessadou are reluctant to communicate with the CIs who inform the Water and Forest Department. Hunters and CIs from Temessadou suggested to inform the official veterinarian if there is a suspected case in the wildlife population whereas hunters from Guendembou suggested to inform the health center.

#### 3.5.4 Strengths and weaknesses of the health information exchange network

The presence of community workers in the three health sectors at the village or district levels is a real asset for a One Health surveillance system. However, they are not systematically informed because of reluctance or ignorance of their role whereas CHHWs and CAHWs are elected by their community. In addition, some community workers do not have telephone or the means to pay telephone credit or to travel the great distance (up to eighteen km) that separates them from the relevant sub-prefectural institutions. These constraints can delay the communication of the health information. CHHWs and CAHWs reported the difficulty to sensitize the community with case definitions and supports for awareness campaigns which are not translated into the Kissi local language. CHHWs required more in-depth training and on more diseases to better detect them. The official veterinarian of Temessadou is overburdened because there are no other veterinarians in this large sub-prefecture and the Fig 5 shows that he could be more solicited by ecosystem health actors for surveillance tasks. CAHWs suggested to train five to six voluntary CAHWs and to officially authorize them to perform specific acts to relieve the official veterinarian. Hunters, CIs and rangers want to participate to a community-based surveillance system but with training on these diseases and on the biosecurity precautions and with protective equipment if they have to handle carcasses. In both sub-prefectures, community workers, community members and breeders compulsory inform local authorities when there is a case of disease. This is an interesting parallel communication channel as the local authorities share the information with the relevant institutions. In return, they are notified if the case is confirmed and provide feedback to their community.

## 4. Discussion

The resurgence of the EVD outbreak from January to June 2021 in Guinea showed improved detection and control capacity compared to the first epidemic in 2014–2016 [48]. This improvement is surely due to the numerous efforts in detection, diagnosis and outbreak response that have been made in the country. However, there are still gaps in the system and efforts still need to be deployed [49]. Our study provides useful insights for strengthening surveillance capacities at a community level, while opting for a tripartite vision of health. We propose recommendations for case definitions and health information exchange network as components of a future community-based surveillance system of emerging zoonotic diseases such as VHFs in Guinea [50]. We believe that an ex ante participatory epidemiology approach improves the performance of the future surveillance system by including contextual factors and local knowledge and empowers communities to define their own solutions to their issues and to take actions [32]. Their participation to this study may increase the acceptability, the feasibility and the sustainability of the future community-based surveillance system. The participatory and One Health approach allowed the integration of actors from the different health sectors (human health, animal health, ecosystem health) that need to be involved in the surveillance of zoonotic diseases such VHFs [51]. The inclusion of the ecosystem health sector in disease surveillance systems is relatively new and we identified potential actors and communication channels [52]. We think that the context of our study area is conducive to the design of a One Health community-based surveillance system because of the actors’ motivation to collaborate, the presence of stakeholders from the three health sectors including community workers [53]. In other contexts, where these conditions are not met, it would be more difficult to implement such a participatory system.

### 4.1 Capacity of the communities to detect diseases

Appropriate case definitions are crucial to ensure the efficiency of the surveillance system, especially for early detection of emerging diseases [54]. A high sensitivity of case definitions will lead to a larger proportion of true positive in the surveillance system, while a low specificity will burden the surveillance system with false positives and will require more investigation resources. If the objective of surveillance is the early detection of emerging zoonotic diseases as serious as the VHFs, a high sensitivity and positive predictive value of the case definitions is desirable [50,55,56]. In low-income countries such as Guinea, which have limited resources for diagnosis and case confirmation, it is also important to avoid false positives as much as possible to not burden the surveillance system. We investigated actors’ knowledge on the diseases to estimate their detection capacity. We also explored actors’ perception of clinical signs to estimate their reaction when they observe one of them and to identify potential signs to include in case definitions. We hypothesized that signs that might constitute an alert, and thus be relevant for inclusion, should be perceived as both rare and health-threatening. The rarity criterion is consistent with a syndromic and community-based surveillance system that aims to early detect emerging diseases [57,58]. We selected the human health threat criterion as an indicator of trigger event for health information communication.

Our results showed that VHFs are poorly described and their associated clinical signs are differently perceived by all human health actors. The controversial aspect of VHFs may have influenced the discussion because it became a taboo issue after the EVD outbreak which caused a tense social and political climate in West Africa [11,59]. This health crisis weakened governments and altered relations between institutions and communities. Communities may be reluctant to report cases for fear of restrictions applied during the epidemic [60]. We did not clearly identify signs of VHFs that are perceived as both rare and health-threatening. Most of them are perceived as frequent and threatening and are not specific to VHFs. Some participants reported to be more threatened by the clinical signs they suffer from or observe in their community. The hemorrhagic signs that are more specific of VHFs are differently perceived among groups. Bleeding is perceived as rare but not threatening by women in both sub-prefectures and red eyes, hematemesis, hematuria and bloody stools are perceived as rare and/or threatening by CHHWs. We suggest to sensitize all the actors to the VHFs’ threat and on the importance to detect them because most of the specific hemorrhagic signs are not perceived as an alert. It could be possible to increase the sensitivity of the case definition by including the unspecific VHFs clinical signs which are perceived as a threat. Except the VHFs, CHHWs showed a better and more homogeneous knowledge of diseases under surveillance than women. None of the interviewed groups perfectly mentioned the clinical pictures included in case definitions but we believe that the training and sensitization of community human health actors had an effect because none of the groups mentioned the diseases under surveillance for which the CHHWs did not have technical sheet with case definitions. Nevertheless, our findings showed that these efforts are insufficient. We suggest to improve training of the CHHWs on diseases under surveillance so that they can all detect them and potentially with more specific case definitions. Awareness efforts should be improved and particularly in Temessadou. We suggest to explore the success key factors of the health program addressing the malaria disease which is well known by communities.

Our results showed that CAHWs have a better knowledge on diseases under surveillance than breeders. We believe that the sensitization and training improved knowledge because the actors better know zoonotic diseases under surveillance than the ones not under surveillance. Nevertheless, sensitization and training efforts should be increased because none of the interviewed groups perfectly mentioned the clinical pictures included in case definitions and some diseases are not mentioned. Our results showed that the clinical signs associated to RVF, that might be interesting to include in case definitions for the surveillance of VHFs in domestic animal populations, are differently perceived among groups. We suggest to create sensitive case definitions including clinical signs perceived as rare or as a threat for human health such as mortality and abortion which are proposed by the FAO for syndromic and participatory surveillance of RVF [47]. We suggest to increase sensitization of animal health actors on the zoonotic risk related to VHFs because they are concerned by the clinical signs they associate with a zoonotic disease ("Everything that is harmful for animal health is harmful for human health", "We are afraid of diseases transmitted from animals to humans.").

Contrary to the human and animal health compartments, the ecosystem sector is not involved in health surveillance at the community level. Knowledge of ecosystem health actors on diseases is almost non-existent but they are able to associate clinical signs to species and to observe abnormalities such lesions on carcasses or one or more animals sick or dead with no apparent cause in the same area. There is no sign perceived both as a threat for human health and rare but all the hemorrhagic signs are perceived as rare. We suggest to include these rare events in a standardized and specific case definition and to train all the ecosystem health actors to zoonotic diseases.

### 4.2 Health information exchange network

We investigated the surveillance network opportunities at the community level. The participants identified the existing communication channels and discussed the strengths and weaknesses of the current information exchange network, their preferences and better alternatives. The results highlighted the fact that community actors face different constraints and communicate with various stakeholders. This situation may delay the report of a suspected case to the relevant institutions and therefore the confirmatory diagnosis and outbreak control actions [51,61]. Our study showed interesting communication channels opportunities in the current health information exchange network to bypass potential barriers to health report. The CHHWs and the CAHWs are a strong link in the information chain because they are geographically close to the community and trained to the surveillance of disease. Therefore, community members and breeders from both sub-prefectures who inform the CHHWs and the CAHWs overcome the lack of transport means that could demotivate them to inform the health center and the official veterinarian but they also potentially reduce the burden on these sub-prefectural actors with not suspected case as the community workers better know the case definitions. Some community members and breeders do not inform the community workers, despite the fact they elected them, because they do not know their role or by reluctance or because they prefer to inform other actors such as matrons, traditional healers and heads of breeders. Promoting the role of community workers and involving other actors who collect health information in the surveillance system may be levers to activate. In the ecosystem health sector any actor is trained to the health surveillance but we identified communication channels opportunities. Because of reluctance hunters prefer to inform the village head of hunters rather than the CIs who are in their immediate area and trained to communicate with Water and Forest Department. The election of the CIs by the community, the promotion of their role and involving heads of hunters in the surveillance system may encourage the health information report to the Water and Forest Department by hunters while avoiding them to travel a great distance and to overwhelm the health center and the official veterinarian with ecosystem health information. One Health collaborations between the health center, the Water and Forest Department and the official veterinarian may be interesting to cross data and detect more efficiently the emergence of a zoonotic disease. They could also share means to confirm cases and jointly notify the existing prefectural One Health unit at the prefectural level rather than notifying each prefectural institution separately [53]. We believe that this study on health communication channels was an essential basis for the future community-based surveillance system. The use of participatory approach created a forum for sharing opinions among actors and may encourage them to collectively design and use adapted health communication channels [62].

### 4.3 Bias and limitations of the study

The major limitation of our study is the low sample size and the resulting potential lack of representativeness of our results. These selection and representativeness bias to our study were due to various reasons [63]. We did not have time to conduct a large number of FGDs and therefore to collect sufficient data for relevant statistical analysis and generalization of results. The participation rate for some FGDs was low and some categories of actors were not interviewed such as traditional healers. We expected to be as representative as possible but the participants convened by our key resource persons in the villages may have be among the most influential of the village. Despite their hierarchical position, we deliberately included heads of breeders and hunters in the FGDs to understand their potential role in the surveillance related to data collection and communication. Women were under-represented and participated less than men during some FGDs. We anticipated this social bias related to gender by conducting non-mixed FGDs. We noted that some activities and occupations are exclusively practiced by men (hunter, breeder) or women (matron). Because of road conditions, we did not visit the most remote villages and therefore we interviewed an unrepresentative sample of the sub-prefectural population. Most of the participants came from the chief towns of the sub-prefectures, where we conducted the FGDs, and neighboring villages. Local knowledge and health communication channels may be different in remote villages as they are further from the human, animal and ecosystem health sub-prefectural institutions and community workers. There is a translation bias to our study as the interviews were conducted in the Kissi language that none of the investigators understood and there may have been approximations or omissions of the information by the translator [64]. We wrote clinical signs and flow diagrams in the Kissi language and we anticipated the fact that some participants were illiterate by using visual tools such clinical signs cards. Due to these constraints, we were not always able to facilitate the participatory tools as originally planned and applied the principle of flexibility to adapt the participatory process to the participants. The investigation team was trained on participatory approaches to avoid professional bias involving interpretation of local knowledge on diseases as veterinarians.

### 4.4 Recommendations

At the end of this study, we recommend to conduct further FGDs with each category of actors we identified as having a potential role in the collection of health information to adapt the case definitions to their knowledge and perception of diseases and clinical signs. Additional FGDs with all the actors are needed to co-design a community health information exchange network and to formalize the One Health collaborations. The implementation of the future community-based surveillance system requires actors training and awareness on case definitions and VHFs; particularly in the ecosystem health sector. Collaboration with the central authorities is essential to ensure the health communication from the community level to the competent institutions for outbreak control. Once implemented we recommend an evaluation of the community-based surveillance system to adjust its components, such as case definitions and health communication channels, to communities’ knowledge and capacities over time. This study could be extended to other regions of Guinea or West Africa that aim to implement a One Health community-based surveillance system of emerging zoonotic diseases.

## Supporting information

S1 Matrix Diseases-clinical signs matrix. Matrix A in S1 Matrix, Human diseases-clinical signs matrix; Matrix B in S1 Matrix, Animal diseases-clinical signs matrix. Guenin, Marie Jeanne, 2022, "Plos NTD research article—A participatory epidemiological and One Health approach for surveillance opportunities", *https*:*//doi*.*org/10*.*18167/DVN1/DBYZXU*, CIRAD Dataverse, V1.
(XLSX)

S2 Matrix Scoring and ranking of clinical signs. Guenin, Marie Jeanne, 2022, "Plos NTD research article—A participatory epidemiological and One Health approach for surveillance opportunities", *https*:*//doi*.*org/10*.*18167/DVN1/DBYZXU*, CIRAD Dataverse, V1.
(XLSX)

S1 Picture Flow diagrams of the information health exchange network. This picture file includes the pictures of flow diagrams representing the health information exchange networks in human health realized by groups of women and community human health workers in Guendembou (Picture A) and Temessadou (Picture B), by breeders and community animal health workers in Guendembou (Picture C) and Temessadou (Picture D), by hunters and community informants in Guendembou (Picture E) and Temessadou (Picture F). Guenin, Marie Jeanne, 2022, "Plos NTD research article—A participatory epidemiological and One Health approach for surveillance opportunities", https://doi.org/10.18167/DVN1/DBYZXU, CIRAD Dataverse, V2.
(PNG)
