# Supplementary figures and images for: A participatory epidemiological and One Health approach to explore the community’s capacity to detect emerging zoonoses and surveillance network opportunities in the forest region of Guinea
Source: PLoS Negl Trop Dis. 2022 Jul 11;16(7):e0010462. doi: 10.1371/journal.pntd.0010462 (PMC9273079; doi:10.1371/journal.pntd.0010462)

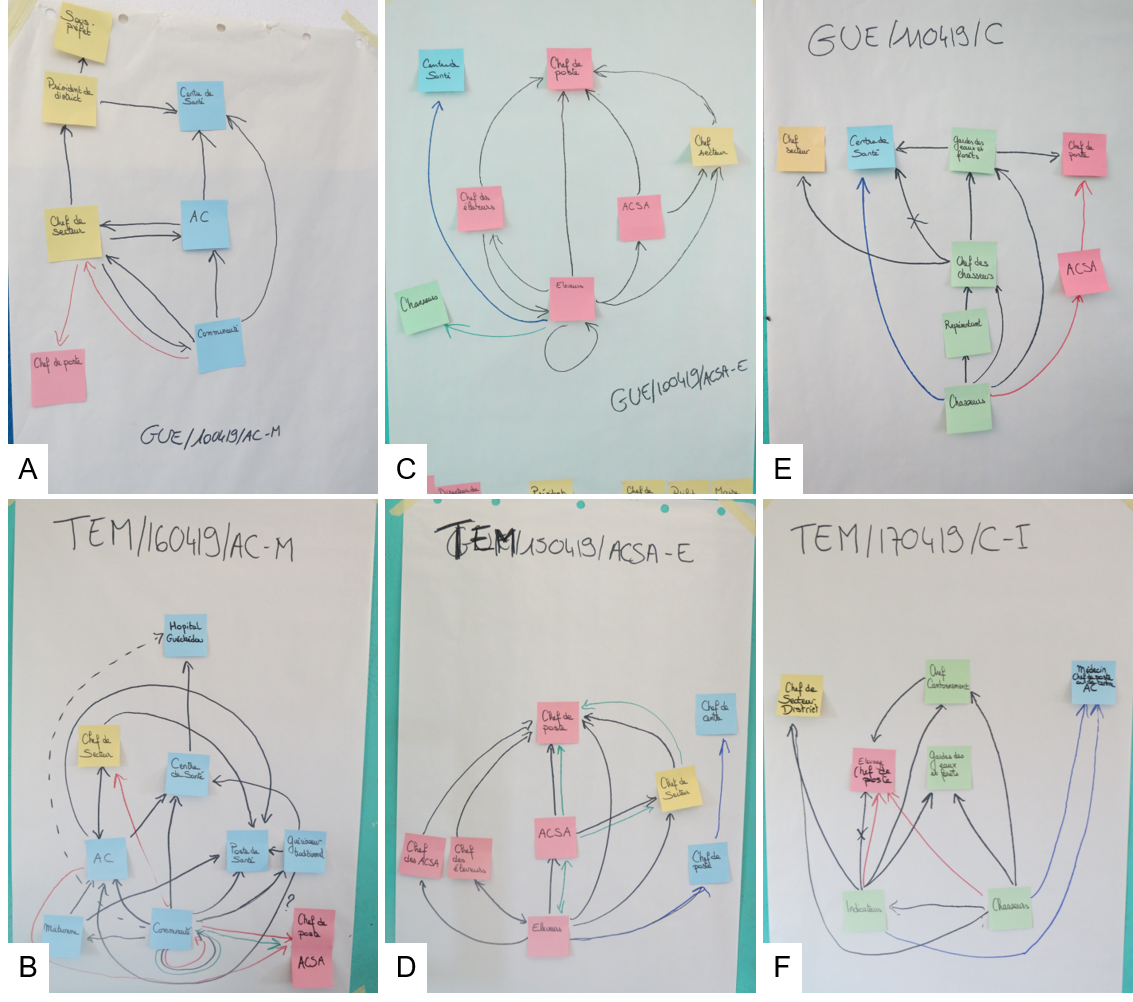

Supplement: S1 Picture — This picture file includes the pictures of flow diagrams representing the health information exchange networks in human health realized by groups of women and community human health workers in Guendembou (Picture A) and Temessadou (Picture B), by breeders and community animal health workers in Guendembou (Picture C) and Temessadou (Picture D), by hunters and community informants in Guendembou (Picture E) and Temessadou (Picture F). Guenin, Marie Jeanne, 2022, "Plos NTD research article—A participatory epidemiological and One Health approach for surveillance opportunities", https://doi.org/10.18167/DVN1/DBYZXU, CIRAD Dataverse, V2. (PNG) [file pntd.0010462.s003.png]
